# A new look towards BAC-based array CGH through a comprehensive comparison with oligo-based array CGH

**DOI:** 10.1186/1471-2164-8-84

**Published:** 2007-03-29

**Authors:** Nicolas Wicker, Annaïck Carles, Ian G Mills, Maija Wolf, Abhi Veerakumarasivam, Henrik Edgren, Fabrice Boileau, Bohdan Wasylyk, Jack A Schalken, David E Neal, Olli Kallioniemi, Olivier Poch

**Affiliations:** 1 Laboratoire de Bioinformatique et de Génomique lntégratives, Institut de Génétique et de Biologie Moléculaire et Cellulaire,1, rue Laurent Fries, BP 10142, 67404 Illkirch CEDEX, France; 2 Uro-Oncology Research Group, Cancer Research UK, Cambridge Research Institute, Li Ka Shing Centre, Robinson Way, Cambridge, CB2 0RE, UK; 3 Medical Biotechnology, VTT Technical Research Centre of Finland and University of Turku, FIN-20520 Turku, Finland; 4 Cancer Research UK Uro-Oncology Research Group, Department of Oncology, University of Cambridge, Hutchison/Medical Research Council Cancer Research Centre, Cambridge CB2 2XZ, England, UK; 5 Human Pathology, Institut de Génétique et de Biologie Moléculaire et Cellulaire,1, rue Laurent Fries, BP 10142, 67404 Illkirch CEDEX, France; 6 Department of Urology (G4-105.1), Academic Medical Centre, University of Amsterdam, Meibergdreef 9, 1105 AZ Amsterdam, The Netherlands

## Abstract

**Background:**

Currently, two main technologies are used for screening of DNA copy number; the BAC (Bacterial Artificial Chromosome) and the recently developed oligonucleotide-based CGH (Chromosomal Comparative Genomic Hybridization) arrays which are capable of detecting small genomic regions with amplification or deletion. The correlation as well as the discriminative power of these platforms has never been compared statistically on a significant set of human patient samples.

**Results:**

In this paper, we present an exhaustive comparison between the two CGH platforms, undertaken at two independent sites using the same batch of DNA from 19 advanced prostate cancers. The comparison was performed directly on the raw data and a significant correlation was found between the two platforms. The correlation was greatly improved when the data were averaged over large chromosomic regions using a segmentation algorithm. In addition, this analysis has enabled the development of a statistical model to discriminate BAC outliers that might indicate microevents. These microevents were validated by the oligo platform results.

**Conclusion:**

This article presents a genome-wide statistical validation of the oligo array platform on a large set of patient samples and demonstrates statistically its superiority over the BAC platform for the Identification of chromosomic events. Taking advantage of a large set of human samples treated by the two technologies, a statistical model has been developed to show that the BAC platform could also detect microevents.

## Background

The study of the genomic imbalances in a variety of different diseases, including cancer, is a major step towards the understanding of disease development. In cancer cells, for example, DNA copy number increases have been shown to be one of the mechanisms by which oncogenes and drug resistance genes can be activated, whereas loss of DNA material may cause inactivation of tumor suppressor genes. Knowledge of copy-number aberrations can have also immediate clinical use in diagnosis and in some cases can provide useful prognostic information. Association of DNA copy-number aberrations with prognosis has been found for a variety of tumor types, including prostate cancer [1], breast cancer [2], gastric cancer [3] and lymphoma [4,5].

Chromosomal Comparative Genomic Hybridization (CGH) is a molecular cytogenetic method for the detection of chromosomal imbalances and it has been extensively used for studying copy number alterations in various cancer types since it was first described in 1992 [6-8]. As classical CGH has an average resolution of 10–20 megabases, it is able to detect changes affecting relatively large chromosomal regions. The introduction of array-based platforms has therefore greatly improved genomic profiling and currently, two technologies are mainly used for screening of DNA copy number; the BAC (Bacterial Artificial Chromosome) and the oligonucleotide-based CGH arrays. BAC-based CGH arrays were amongst the first genomic arrays to be introduced [9] and are routinely used to detect single copy changes in the genome, owing to their high sensitivity. However, producing BAC clones for array CGH (aCGH) is expensive and time-consuming and, due to the large size of BACs, the limits of BAC aCGH resolution have been reached. Oligonucleotide aCGH [10,11] allow flexibility in probe design, greater coverage, and much higher resolution. The latter depends on array design and the cell type homogeneity. Moreover, oligonucleotides can more easily be produced for any organism for which the genome has been sequenced. Today, as noted in [12], the aCGH field is evolving towards oligonucleotide aCGH.

IJssel [13] has published a genome-wide validation of their in-house spotted oligo aCGH using BAC arrays, for human and mouse samples. These samples consist of one human gastric tumour and two different mouse tumours, and the comparison between BAC and oligo aCGH was performed on the genomic profiles. In this study, IJssel distinguished two kinds of noise, the technical noise and the true genomic copy number polymorphisms [14]. Various algorithms for data denoising exist such as the non-parametric method called circular binary segmentation (CBS) [15] which splits the chromosomes into regions of equal copy number. CBS is identified as one of the best segmentation methods [16].

Here, we present a comprehensive comparison between the two aCGH platforms using available data from 19 human advanced prostate cancer (European PRIMA project, PRostate cancer Integral Management Approach) obtained at two independent sites with the same batch of DNA. This comprehensive comparison has been performed on raw data sets and data analysed using CBS [15]. This represents the first genome-wide statistical validation of the oligo array platform. In addition we have developed a statistical method to identify BAC outliers that could represent microevents.

## Results

### Probe distribution and noise levels for the BAC and oligo platforms

The repartitions of BACs and oligos on the human genome were first compared. The uniformity of the 3040 BACs and the 40319 oligos repartition along the human genome was tested using the Kolmogorov-Smirnov test (p-value < 0.01), considering each chromosome arm as an interval and each BAC and oligo as a point. BACs are uniformly distributed along all the chromosomes whereas oligos are not, except on chromosome 18 and arms 7p, 10p and 19p.

**Figure 1:**
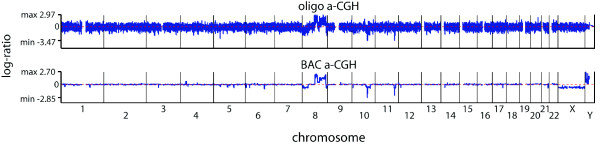
**Genomic data display for BAC and oligo aCGH data**. The bottom plot represents BAC aCGH data on the human genome and the upper one the oligo aCGH data for the same patient sample (819). Red dotted-line on the two aCGH plots indicates two copies. The sex mismatch was performed for the BAC aCGH but not for the oligo.

Figure 1 illustrates the data from both platforms for the same patient sample (819) along the human genome. Both platforms detect aberrations, for instance on chromosomes 8, 10 and 11, and the log-ratios of both platforms oscillate around stable values. To measure the noise, we have computed the autocorrelation of the log-ratios inside each chromosome and found 0.36 and 0.18 for BAC and oligo aCGH data, respectively. This implies that there is more baseline Variation in oligo-based aCGH data compared to BAC aCGH data.

### A log-ratio comparison of the oligo and BAC CGH platforms

**Figure 2:**
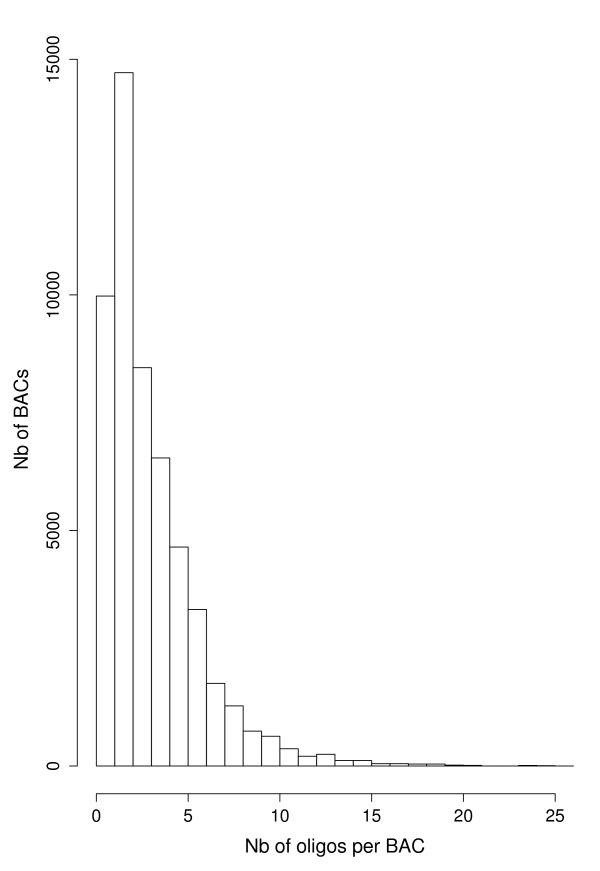
**Distribution of the number of oligos per BAC for all the 19 patient samples**. 9975 BACs have no oligos and 1345 have at least 10 oligos.

**Figure 3:**
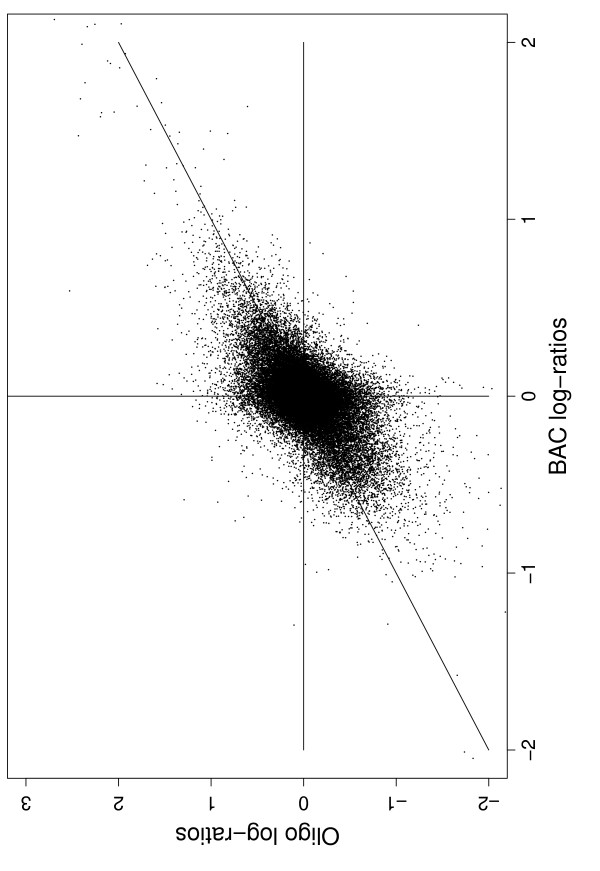
**Dotplot of BAC log-ratios versus oligo log-ratios**. The dot-plot has been done for the 43372 BACs of the 19 patients having at least one corresponding oligo.

The comparison was restricted to the 22 autosomes as the available data were produced with the sex mismatch for BAC aCGH and not for oligo aCGH. Each BAC value was compared with the log-ratios of the corresponding oligos, i.e oligos positioned between the start and the end of the BAC. This comparison is not straight-forward as the mean number of oligos per BAC across the 19 patients is only 2.5. The details of the distribution of the number of oligos per BAC are shown in figure 2. A one-sample Student test was performed for the 1345 BACs that overlap with at least 10 oligos, so that the test had enough power. There were 32 BAC log-ratios significantly different from their corresponding oligo log-ratios (p-value < 0.01). To provide a more global comparison, a complementary analysis was carried out on the 43372 BAC values corresponding to at least one oligo in the 19 patients. The comparison was done by Computing the Kendall correlation for all pairs of BAC and oligo log-ratios, the oligo value being a mean oligo log-ratio. The pair values are plotted on figure 3. The correlation gives a significant p-value inferior to 1*e *- 15 with *τ *= 0.37. A *τ *value of 0.37 indicates that both values of a pair increase or decrease with a probability of 68.5%. When the Kendall's correlation test is performed excluding BACs containing fewer than 6 oligos, *τ *reaches 0.44. This comparison shows that there is a significant correlation between the BAC and the oligo platform results.

### Comparison between oligo and BAC CGH using segmentation

**Figure 4:**
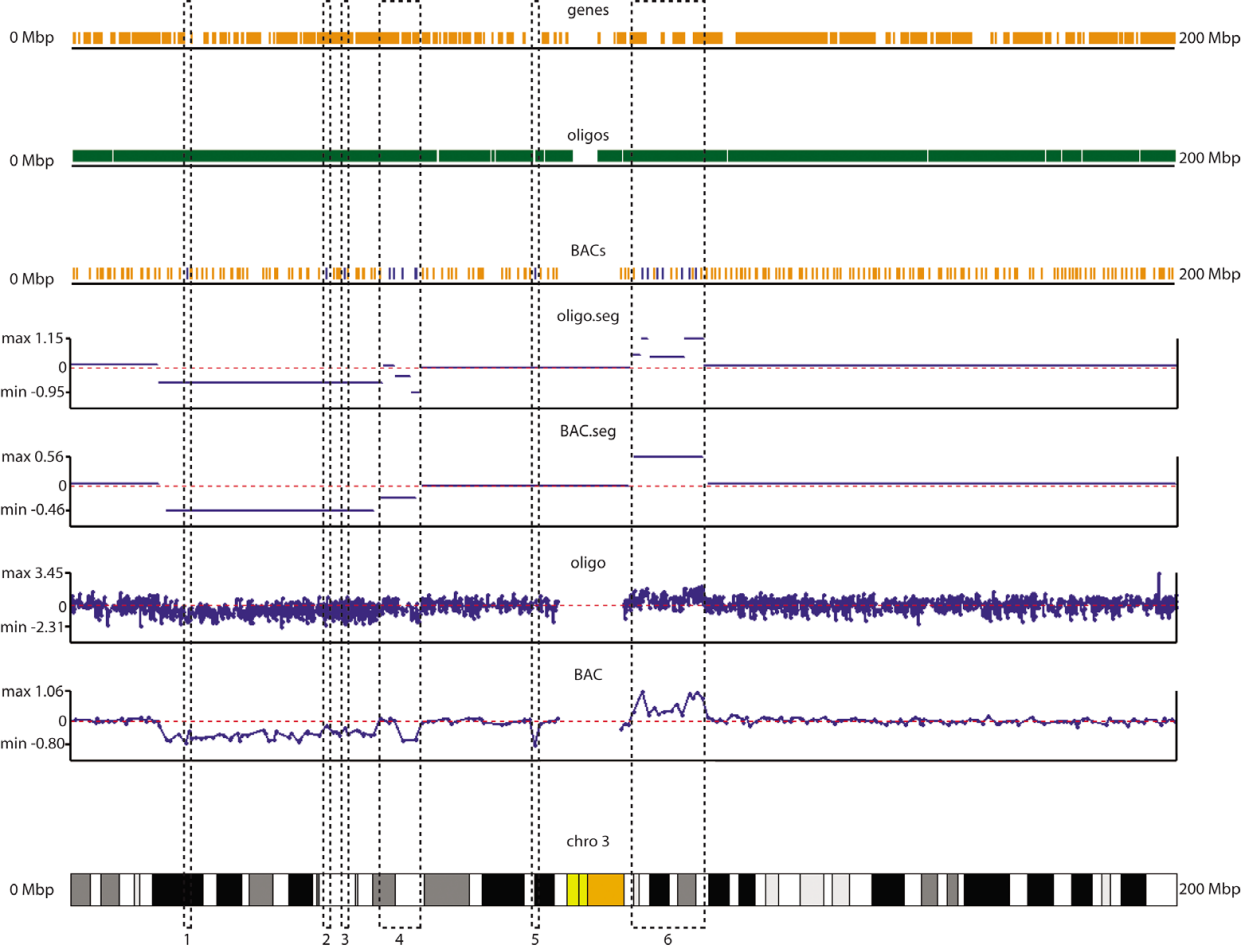
**Chromosomic data display for BAC aCGH data and oligo aCGH data**. From bottom to top are represented on chromosome 3 of patient sample 817, the BAC aCGH data plot, the oligo aCGH data plot, the segmented BAC data plot, the segmented oligo data plot, the CGH BACs (in blue are represented the BAC outliers), the CGH oligonucleotides and the genes. The black dotted boxes indicate the different regions of detected BAC outliers (1, 2, 3, 4, 5 and 6).

Oligo and BAC CGH platforms were compared after segmentation into regions of equal copy number as the individual number of copies can be corrupted with noise. The Circular Binary Segmentation method (CBS) [15] was used both for BAC and oligo data. This is illustrated in figure 4 for chromosome 3 from patient 817.

**Figure 5:**
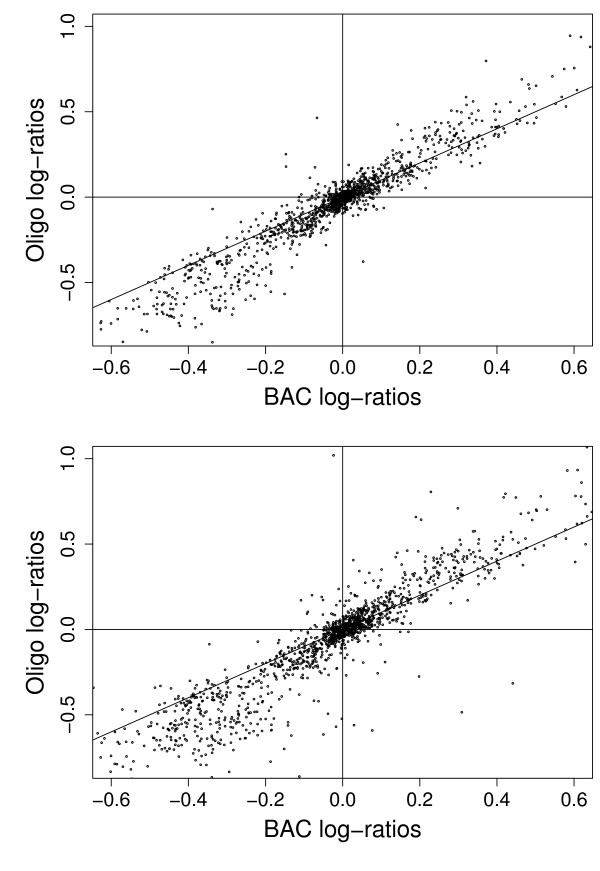
**Global comparison between BAC and oligo Segments**. The reference segments obtained by CBS [15] are BACs on the top plot and oligo on the bottom.

A statistical comparison was performed to assess the divergence of the BAC and oligo data in the regions delimited by the segmentation. Each segment is considered as a log-ratios sample and two comparisons were made. Firstly, we compared globally all the BAC and oligo Segments. Each BAC segment was compared with the corresponding oligos, located between the start and the end of the BAC segment. This was also done reciprocally with the oligo Segments as the starting point, as the comparison is asymmetrical. The pair segment mean values of all patient samples are shown in figure 5. The Kendall correlation gives a p-value inferior to 1*e *- 15, with *τ *significantly greater (0.81 and 0.80 for BAC and oligo Segments respectively) than the value of 0.37 achieved without segmentation.

Secondly, all the segments were compared individually. Before comparing the segment samples, a Shapiro test was performed to determine whether their distributions are gaussian. A Student test was used to compare the Gaussian distributions, otherwise a Wilcoxon-Mann-Whitney test was applied. A 1% false discovery rate [17] was then used to avoid problems related to multiple testing. Using these approaches we have identified 173 of 1231 (14%) BAC segments with values divergent from oligo values (BAC versus oligo) and 177 of 1560 (11%) oligo divergent segments (oligo versus BAC). To complete this result the same comparison was done with a false discovery rate of 5% and 10%. For 5% the number of divergent segments are then 272 and 338 for BAC versus oligo and oligo versus BAC respectively and for 10%, 299 and 375.

Finally, the comparison of the two platforms was focused on the detection of aberrant events. We considered as aberrations all segments with mean number of copies lower than 1.5 or higher than 2.5. The BAC platform detected 71 deletions and 74 amplifications among the 19 patient samples whereas the oligo platform detected 314 deletions and 209 amplifications with median lengths of 12 Mbp and 3.5 Mbp respectively. This shows that oligo aCGH detect more, smaller chromosomal events than BAC aCGH. Regarding the genomic location of these segments, 40 segments were found by both platforms either deleted or amplified with an overlapping region comprising more than 90% of each segment. It is important to note that their median length is 27 Mbp, indicating that large chromosomal aberrations are found by both platforms.

In conclusion, segmentation enhances the correlation between the two platforms. Another result is that the oligo aCGH reveal many more, smaller aberrations.

### Detection of outliers in BAC segments

In order to perform a more in-depth analysis of both platforms, that could then be applied to the identification of target genes and regions of genetic alteration, we developed a statistical model to detect BAC outliers and validated the results with the oligo CGH platform. An advantage of the oligo CGH platform is its ability to detect microevents. In this section we show that, to some extent, the BAC aCGH platform, and more precisely BAC outliers within segments, could indicate microevents.

BAC outliers can be detected by modelling their log-ratios with a Gaussian distribution *N*(*μ*_*s*_, *σ*) where *μ*_*s *_is the theoretical mean of the BAC segment *s *and *σ *the Standard deviation across all segments and all patients. However to avoid misestimating *σ *by including too many outliers, this parameter has been estimated on the least varying patient, 812, containing *n *= 3017 BACs. Let μ^
 MathType@MTEF@5@5@+=feaafiart1ev1aaatCvAUfKttLearuWrP9MDH5MBPbIqV92AaeXatLxBI9gBaebbnrfifHhDYfgasaacH8akY=wiFfYdH8Gipec8Eeeu0xXdbba9frFj0=OqFfea0dXdd9vqai=hGuQ8kuc9pgc9s8qqaq=dirpe0xb9q8qiLsFr0=vr0=vr0dc8meaabaqaciaacaGaaeqabaqabeGadaaakeaaiiGacuWF8oqBgaqcaaaa@2E79@_*s *_and σ^
 MathType@MTEF@5@5@+=feaafiart1ev1aaatCvAUfKttLearuWrP9MDH5MBPbIqV92AaeXatLxBI9gBaebbnrfifHhDYfgasaacH8akY=wiFfYdH8Gipec8Eeeu0xXdbba9frFj0=OqFfea0dXdd9vqai=hGuQ8kuc9pgc9s8qqaq=dirpe0xb9q8qiLsFr0=vr0=vr0dc8meaabaqaciaacaGaaeqabaqabeGadaaakeaaiiGacuWFdpWCgaqcaaaa@2E86@ be the estimators of *μ*_*s *_and *σ*. To detect outliers the following classical calculus was performed considering each BAC value *x *inside its corresponding segment *s *of size *n*_*s*_. For each BAC, *μ *was reestimated by Computing a new *μ*_*s *_on the *n*_*s *_- 1 value left after excluding the *x *value.

x−μ^s=x−μs−(μ^s−μs)⇔ns−1(x−μ^s)σ=ns−1(x−μs)σ−ns−1(μ^s−μs)σ
 MathType@MTEF@5@5@+=feaafiart1ev1aaatCvAUfKttLearuWrP9MDH5MBPbIqV92AaeXatLxBI9gBaebbnrfifHhDYfgasaacH8akY=wiFfYdH8Gipec8Eeeu0xXdbba9frFj0=OqFfea0dXdd9vqai=hGuQ8kuc9pgc9s8qqaq=dirpe0xb9q8qiLsFr0=vr0=vr0dc8meaabaqaciaacaGaaeqabaqabeGadaaakeaacqWG4baEcqGHsisliiGacuWF8oqBgaqcamaaBaaaleaacqWGZbWCaeqaaOGaeyypa0JaemiEaGNaeyOeI0Iae8hVd02aaSbaaSqaaiabdohaZbqabaGccqGHsislcqGGOaakcuWF8oqBgaqcamaaBaaaleaacqWGZbWCaeqaaOGaeyOeI0Iae8hVd02aaSbaaSqaaiabdohaZbqabaGccqGGPaqkcqGHuhY2daWcaaqaamaakaaabaGaemOBa42aaSbaaSqaaiabdohaZbqabaGccqGHsislcqaIXaqmaSqabaGccqGGOaakcqWG4baEcqGHsislcuWF8oqBgaqcamaaBaaaleaacqWGZbWCaeqaaOGaeiykaKcabaGae83Wdmhaaiabg2da9maalaaabaWaaOaaaeaacqWGUbGBdaWgaaWcbaGaem4CamhabeaakiabgkHiTiabigdaXaWcbeaakiabcIcaOiabdIha4jabgkHiTiab=X7aTnaaBaaaleaacqWGZbWCaeqaaOGaeiykaKcabaGae83WdmhaaiabgkHiTmaalaaabaWaaOaaaeaacqWGUbGBdaWgaaWcbaGaem4CamhabeaakiabgkHiTiabigdaXaWcbeaakiabcIcaOiqb=X7aTzaajaWaaSbaaSqaaiabdohaZbqabaGccqGHsislcqWF8oqBdaWgaaWcbaGaem4CamhabeaakiabcMcaPaqaaiab=n8aZbaaaaa@74B2@

As ns−1(x−μs)σ
 MathType@MTEF@5@5@+=feaafiart1ev1aaatCvAUfKttLearuWrP9MDH5MBPbIqV92AaeXatLxBI9gBaebbnrfifHhDYfgasaacH8akY=wiFfYdH8Gipec8Eeeu0xXdbba9frFj0=OqFfea0dXdd9vqai=hGuQ8kuc9pgc9s8qqaq=dirpe0xb9q8qiLsFr0=vr0=vr0dc8meaabaqaciaacaGaaeqabaqabeGadaaakeaadaWcaaqaamaakaaabaGaemOBa42aaSbaaSqaaiabdohaZbqabaGccqGHsislcqaIXaqmaSqabaGccqGGOaakcqWG4baEcqGHsisliiGacqWF8oqBdaWgaaWcbaGaem4CamhabeaakiabcMcaPaqaaiab=n8aZbaaaaa@3B00@ and ns−1(μ^s−μs)σ
 MathType@MTEF@5@5@+=feaafiart1ev1aaatCvAUfKttLearuWrP9MDH5MBPbIqV92AaeXatLxBI9gBaebbnrfifHhDYfgasaacH8akY=wiFfYdH8Gipec8Eeeu0xXdbba9frFj0=OqFfea0dXdd9vqai=hGuQ8kuc9pgc9s8qqaq=dirpe0xb9q8qiLsFr0=vr0=vr0dc8meaabaqaciaacaGaaeqabaqabeGadaaakeaadaWcaaqaamaakaaabaGaemOBa42aaSbaaSqaaiabdohaZbqabaGccqGHsislcqaIXaqmaSqabaGccqGGOaakiiGacuWF8oqBgaqcamaaBaaaleaacqWGZbWCaeqaaOGaeyOeI0Iae8hVd02aaSbaaSqaaiabdohaZbqabaGccqGGPaqkaeaacqWFdpWCaaaaaa@3CED@ obey asymptotically the *N*(0, ns−1
 MathType@MTEF@5@5@+=feaafiart1ev1aaatCvAUfKttLearuWrP9MDH5MBPbIqV92AaeXatLxBI9gBaebbnrfifHhDYfgasaacH8akY=wiFfYdH8Gipec8Eeeu0xXdbba9frFj0=OqFfea0dXdd9vqai=hGuQ8kuc9pgc9s8qqaq=dirpe0xb9q8qiLsFr0=vr0=vr0dc8meaabaqaciaacaGaaeqabaqabeGadaaakeaadaGcaaqaaiabd6gaUnaaBaaaleaacqWGZbWCaeqaaOGaeyOeI0IaeGymaedaleqaaaaa@31AE@) and *N*(0,1) law respectively, the distribution of ns−1(x−μ^s)σ
 MathType@MTEF@5@5@+=feaafiart1ev1aaatCvAUfKttLearuWrP9MDH5MBPbIqV92AaeXatLxBI9gBaebbnrfifHhDYfgasaacH8akY=wiFfYdH8Gipec8Eeeu0xXdbba9frFj0=OqFfea0dXdd9vqai=hGuQ8kuc9pgc9s8qqaq=dirpe0xb9q8qiLsFr0=vr0=vr0dc8meaabaqaciaacaGaaeqabaqabeGadaaakeaadaWcaaqaamaakaaabaGaemOBa42aaSbaaSqaaiabdohaZbqabaGccqGHsislcqaIXaqmaSqabaGccqGGOaakcqWG4baEcqGHsisliiGacuWF8oqBgaqcamaaBaaaleaacqWGZbWCaeqaaOGaeiykaKcabaGae83Wdmhaaaaa@3B10@ is the *N*(0, ns
 MathType@MTEF@5@5@+=feaafiart1ev1aaatCvAUfKttLearuWrP9MDH5MBPbIqV92AaeXatLxBI9gBaebbnrfifHhDYfgasaacH8akY=wiFfYdH8Gipec8Eeeu0xXdbba9frFj0=OqFfea0dXdd9vqai=hGuQ8kuc9pgc9s8qqaq=dirpe0xb9q8qiLsFr0=vr0=vr0dc8meaabaqaciaacaGaaeqabaqabeGadaaakeaadaGcaaqaaiabd6gaUnaaBaaaleaacqWGZbWCaeqaaaqabaaaaa@2FBC@) law. Besides, it is known that the law of nσ^2σ
 MathType@MTEF@5@5@+=feaafiart1ev1aaatCvAUfKttLearuWrP9MDH5MBPbIqV92AaeXatLxBI9gBaebbnrfifHhDYfgasaacH8akY=wiFfYdH8Gipec8Eeeu0xXdbba9frFj0=OqFfea0dXdd9vqai=hGuQ8kuc9pgc9s8qqaq=dirpe0xb9q8qiLsFr0=vr0=vr0dc8meaabaqaciaacaGaaeqabaqabeGadaaakeaadaWcaaqaaiabd6gaUHGaciqb=n8aZzaajaWaaWbaaSqabeaaieaacqGFYaGmaaaakeaacqWFdpWCaaaaaa@32E1@ is asymptotically χn−12
 MathType@MTEF@5@5@+=feaafiart1ev1aaatCvAUfKttLearuWrP9MDH5MBPbIqV92AaeXatLxBI9gBaebbnrfifHhDYfgasaacH8akY=wiFfYdH8Gipec8Eeeu0xXdbba9frFj0=OqFfea0dXdd9vqai=hGuQ8kuc9pgc9s8qqaq=dirpe0xb9q8qiLsFr0=vr0=vr0dc8meaabaqaciaacaGaaeqabaqabeGadaaakeaaiiGacqWFhpWydaqhaaWcbaGaemOBa4MaeyOeI0IaeGymaedabaGaeGOmaidaaaaa@32CB@. From this it is inferred that: t=ns−1(x−μ^s)nsσnσ^2(n−1)σ2
 MathType@MTEF@5@5@+=feaafiart1ev1aaatCvAUfKttLearuWrP9MDH5MBPbIqV92AaeXatLxBI9gBaebbnrfifHhDYfgasaacH8akY=wiFfYdH8Gipec8Eeeu0xXdbba9frFj0=OqFfea0dXdd9vqai=hGuQ8kuc9pgc9s8qqaq=dirpe0xb9q8qiLsFr0=vr0=vr0dc8meaabaqaciaacaGaaeqabaqabeGadaaakeaacqWG0baDcqGH9aqpdaWcaaqaamaalaaabaWaaOaaaeaacqWGUbGBdaWgaaWcbaGaem4CamhabeaakiabgkHiTiabigdaXaWcbeaakiabcIcaOiabdIha4jabgkHiTGGaciqb=X7aTzaajaWaaSbaaSqaaiabdohaZbqabaGccqGGPaqkaeaadaGcaaqaaiabd6gaUnaaBaaaleaacqWGZbWCaeqaaaqabaGccqWFdpWCaaaabaWaaOaaaeaadaWcaaqaaiabd6gaUjqb=n8aZzaajaWaaWbaaSqabeaacqaIYaGmaaaakeaacqGGOaakcqWGUbGBcqGHsislcqaIXaqmcqGGPaqkcqWFdpWCdaahaaWcbeqaaiabikdaYaaaaaaabeaaaaaaaa@4CFE@ obeys asymptotically a Student's law *T*_*n*-1_. *t *is simplified to : t=x−μ^sσ^(ns−1)(n−1)nsn
 MathType@MTEF@5@5@+=feaafiart1ev1aaatCvAUfKttLearuWrP9MDH5MBPbIqV92AaeXatLxBI9gBaebbnrfifHhDYfgasaacH8akY=wiFfYdH8Gipec8Eeeu0xXdbba9frFj0=OqFfea0dXdd9vqai=hGuQ8kuc9pgc9s8qqaq=dirpe0xb9q8qiLsFr0=vr0=vr0dc8meaabaqaciaacaGaaeqabaqabeGadaaakeaacqWG0baDcqGH9aqpdaWcaaqaaiabdIha4jabgkHiTGGaciqb=X7aTzaajaWaaSbaaSqaaiabdohaZbqabaaakeaacuWFdpWCgaqcaaaadaGcaaqaamaalaaabaGaeiikaGIaemOBa42aaSbaaSqaaiabdohaZbqabaGccqGHsislcqaIXaqmcqGGPaqkcqGGOaakcqWGUbGBcqGHsislcqaIXaqmcqGGPaqkaeaacqWGUbGBdaWgaaWcbaGaem4Camhabeaakiabd6gaUbaaaSqabaaaaa@4700@. A p-value is computed for each BAC using the following procedure:

   For each segment do

      For each BAC inside current segment do

         Produce a sample of all the segment log-ratios except the current BAC one

         Compute a p-value for the BAC value *x *according to formula

         P(Tn−1≥x−μ^sσ^(ns−1)(n−1)nsn)
 MathType@MTEF@5@5@+=feaafiart1ev1aaatCvAUfKttLearuWrP9MDH5MBPbIqV92AaeXatLxBI9gBaebbnrfifHhDYfgasaacH8akY=wiFfYdH8Gipec8Eeeu0xXdbba9frFj0=OqFfea0dXdd9vqai=hGuQ8kuc9pgc9s8qqaq=dirpe0xb9q8qiLsFr0=vr0=vr0dc8meaabaqaciaacaGaaeqabaqabeGadaaakeaacqWGqbaudaqadaqaaiabdsfaunaaBaaaleaacqWGUbGBcqGHsislcqaIXaqmaeqaaOGaeyyzIm7aaSaaaeaacqWG4baEcqGHsisliiGacuWF8oqBgaqcamaaBaaaleaacqWGZbWCaeqaaaGcbaGaf83WdmNbaKaaaaWaaOaaaeaadaWcaaqaaiabcIcaOiabd6gaUnaaBaaaleaacqWGZbWCaeqaaOGaeyOeI0IaeGymaeJaeiykaKIaeiikaGIaemOBa4MaeyOeI0IaeGymaeJaeiykaKcabaGaemOBa42aaSbaaSqaaiabdohaZbqabaGccqWGUbGBaaaaleqaaaGccaGLOaGaayzkaaaaaa@4DB4@

      Done

   Done

All p-values are treated according to [17] taking *FDR *= 1%.

This model was used on the 19 patient samples. 990 outliers were detected in the genomic profiles of the patients, across all the chromosomes. Figure 4 shows 4 isolated BAC outliers (boxes 1, 2, 3 and 5) and two groups of BAC outliers (boxes 4 and 6) on chromosome 3 of patient sample 817. Isolated BAC outliers point to putative microevents (microdeletion or small amplification) whereas groups of BAC outliers may indicate larger aberred regions. Finally to validate statistically this result, the Kendall's correlation with oligos was computed for the 749 BAC outliers with at least one corresponding oligo giving *τ *= 0.54 and a p-value of 1*e *- 15.

## Discussion

The conceptual advantage of a high density oligo aCGH is that it can reveal microdeletions or amplifications at the gene level that may contribute to gene transcript Variation and that are not detected on a BAC platform. Indeed the oligo platform has an average 35 kbp spatial resolution that enables to span all the well characterised genes (defined in NCBI build 35, May 2004) providing sufficient coverage for a genome wide survey of DNA aberrations. Different sizes of microdeletions, from 2 to 7 probes have been detected by the CBS method, such as the microdeletion containing the potential tumor suppressor ATBF1 [18].

In this paper, oligo aCGH results are validated using the 3 k BAC aCGH platform on data from 19 patients. The clinical material used in the PRIMA project is extremely unusual, displaying greater than 75% tumour cellularity. In general, other than in very advanced cancers, the level of tumour cellularity would be significantly lower for prostatic material and this together with lymphocytic infiltration might be expected to add to the noise component of the oligo aCGH signal and reduce concordance both with BAC array data and gene expression microarrays. The number of patients involved in this experiment allowed us to perform statistically significant analyses. Indeed there are 1345 BACs that overlap with at least 10 oligos and 43372 BACs with at least one oligo in the 19 patients.

The validation was first carried out directly, by matching each BAC to the corresponding oligo raw data, and then indirectly, using a segmentation algorithm called CBS [15], which gave more significant results.

The Kendall's correlation on the raw data was significant, with *τ *= 0.37, and was improved by segmentation, reaching a value of 0.8. Correlation was therefore increased more than two fold when compared to the direct comparison, confirming the observation of IJssel [13], that BAC and oligo profiles are very similar after smoothing. This global analysis of all BAC and oligo Segments was complemented by an individual comparison, where each segment from BAC or oligo platforms was tested against its corresponding oligo or BAC data set. 14% and 11% of BAC and oligo segments respectively were found to be divergent. These percentages can be lowered (10% and 7%) by scaling the oligo values as the log-ratios are higher for oligos than for BACs again in agreement with IJssel [13] (figure 5). However these statistical tests only reveal very divergent segments, indeed taking false discovery rates equal to 5 or 10% gives higher percentages (22% and 19%, 27% and 22% respectively). So this number of divergent segments should be regarded as a lower bound. Indeed there were only few similar copy number aberrations between the two platforms as the oligo platform presented the advantage to detect more, smaller chromosomic aberrations. Secondly, we compared the noise level between the two platforms on raw and segmented data. Computing the autocorrelation along each chromosome, we observed that the baseline variation for BACs is lower than for oligos in agreement with previous studies by IJssel [13] and Ylstra [12] who both computed the standard deviation on regions without copy number changes. Using the regions of equal copy numbers produced by CBS, the means of the oligo and BAC segments standard deviations have been computed giving respectively 0.32 and 0.10 confirming the previous result. BACs with large insert clones display a lower variation compared with the oligo platform. However, the trade-off is a lower sensibility for BACs compared to oligos. Besides, the standard deviation value of 0.32 of the oligo platform, is small from a statistical point of view. Indeed, the confidence interval of the true number of copies inside segments is small. For instance for a segment with 10 oligos inside, a standard deviation of 0.32 means that for a mean value of 3 copies, the interval is 2.5 – 3.6 (probability = 99%).

In spite of the many advantages of the oligo aCGH platform, there is still some value in using the BAC platform. BACs are distributed uniformly on the human genome so that regions not previously found to be involved in cancer or non-coding regions are covered. This *terra incognita *may be interesting for further investigation, in particular to search for microRNAs and repeated sequences. In addition, a BAC outlier detection model that could point to putative microevents has been introduced in this article. The BAC outlier values have been compared with the corresponding oligo values with good correlation. A large number of these BAC outliers (35%) were found in regions without known genes according to UCSC (June 2005). However these BAC outliers represent large regions of 150 kbp and the potential microevents must still be precisely located and biologically validated.

## Conclusion

We have performed a large scale comparison of oligo and BAC platforms using a set of 19 patient samples. First, we have established statistically the reliability of the oligo platform for the identification of chromosomic events. Moreover the oligo platform outperforms the BAC technology for the detection of more, smaller aberrations. Taking advantage of this large set of data, we have developped a statistical model, that highlights that BACs may detect putative microevents. Hopefully, this result will incite researchers to reconsider the potential use of BAC data for more in-depth investigation of new data, as well as for the numerous publically available BAC CGH data. The challenge in future studies will be the routine establishment of banks of well-defined laser captured material, so that the greater sensitivity of these platforms can be successfully exploited. In order to obtain a more complete picture of cancer, attempts could then be made to combine the aCGH approaches with transcriptomic and proteomic technologies.

## Methods

### Prostate cancer samples and DNA extraction

The analyzed sample set is composed of 19 advanced prostate cancer samples from 18 patients. Frozen tissue blocks were step-sectioned using a cryostat, and 20 *μ*m sections were collected in frozen tubes for subsequent DNA and RNA extractions.

### Isolation of DNA

DNA was extracted from the samples after overnight proteinase K treatment using standard protocols [19]. For all samples, the same batch of DNA was used for both BAC and oligo aCGH analyses to make the data comparable.

### BAC aCGH plateform

BAC clones were selected from RP-1, RP-5 and RP-6, RP-11 [20] and CalTech BAC [21]. The clones were selected from the published Golden Path and spaced at approximately 1-Mb intervals across the arm of each chromosome. Drosophila BACs were obtained from the RPCI-98 library [20]. All clones were screened for T1 phage contamination, streaked to a single colony, and verified by fingerprinting. Clone details can be obtained from the Ensembl database accessed in the CytoView pages [22]. The resultant 1 Mb whole-genome BAC aCGH has been previously described [19].

DNA was labeled by use of a Bioprime Labeling Kit (Invitrogen, Carlsbad, CA) with modification of the nucleotide mix. Briefly, a 84 *μ*l reaction was set up containing 600 ng of DNA and a final concentration of 1 × Random Primers Solution. After denaturing the DNA for 10 min at 100°C, 10 *μ*l l0 × dNTP mix (0.5 mM dCTP, 2 mM dATP, 2 mM dGTP, 2 mMdTTP in TE-buffer), 4 *μ*l 1 mM Cy5-dCTP or Cy3-dCTP (NEN Life Science Products, Boston, MA) and 2 *μ*l Klenow fragment supplied in the kit were added on ice to produce a final reaction volume of 100 *μ*l. The reaction was incubated at 37°C overnight and stopped by adding 10 *μ*l stop buffer (Bioprime Labeling Kit; Invitrogen). Unincorporated nucleotides were removed by use of microspin G50 columns (Pharmacia Biotech, Piscataway, NJ) according to the instruction of the suppliers.

The arrays have an area 3 × 2 cm. Female genomic DNA was used as reference. Test and reference DNA (180 *μ*l each) were combined, precipitated together with 135 *μ*l of human Cot1 DNA (Invitrogen), and resuspended in 60 *μ*l of hybridization buffer (50% formamide, 10% dextran sulfate, 0.1% Tween 20, 2 SSC, and 10 mM Tris/HCl, pH 7.4) and 3 *μ*l of yeast tRNA (100 *μ*g/*μ*l; Invitrogen). A ring of rubber cement was closely applied around the array to form a well. After denaturing the sample for 10 min at 72°C, the denatured herring sperm Cot1 DNA mix was added and the array incubated in a humidity chamber containing 3 MM paper (Whatmann, Hillsboro, OR) saturated with 2 × SSC and 40% formamide on a table rocking at 5 rpm at 37°C for 60 min. Arrays were prehybridized as follows: 80 *μ*l of herring sperm DNA (10 mg/ml; Sigma) and 135 *μ*l of human Cot1 DNA (Invitrogen) were precipitated, resuspended in 80 *μ*l of hybridization buffer, and denatured for 10 min at 72°C. The prehybridization solution was then removed and replaced by the prehybridized genomic DNA. The slide was transferred into a small hybridization chamber containing Whatmann 3 MM paper saturated with 2 × SSC and 20% formamide, sealed with parafilm, and incubated on a rocking table (5 rpm) at 37°C for 48 hr. Slides were washed for 10 min at room temperature in PBS 0.05% Tween 20, 30 min at 42°C in 50% formamide 2 × SSC, and 10 min at room temperature in PBS 0.05% Tween 20, before being dried by spinning in a centrifuge for 5 min at 150 g and stored until scanning.

Arrays were scanned by use of an Axon 4000B scanner (Axon Instruments, Burlingame, CA). Images were analyzed by use of GenePix Pro 3.0 software (Axon Instruments). Spots were defined by use of the automatic grid feature of the software and manually adjusted where necessary. Fluorescence intensities of all spots were then calculated after subtraction of local background. To correct for non-specific hybridization to spotted DNA, the mean intensity of all of the Drosophila clones was subtracted for each fluorochrome from each of the human clones before ratio calculation (Drosophila correction).

### Oligo aCGH platform

Oligonucleotide aCGH was performed according to the protocol provided by Agilent Technologies (oligonucleotide aCGH for genomic DNA analysis, protocol version 2.0, August 2005, Agilent Technologies, Palo Alto, CA), with minor modifications. Briefly, 12 *μ*g of genomic DNA was digested overnight with AluI and RsaI followed by purification using phenol-chloroform extraction. Male genomic DNA (catalog number G1471, Promega, Madison, WI) was used as reference in all hybridizations in order to analyse the chromosome X as its related copy number alterations play a key role in prostate cancer. 3 *μ*g of digested tumour DNA and reference DNA was labeled with Cy5-dUTP and Cy3-dUTP (PerkinElmer, Wellesley, MA), respectively, in a random priming reaction using Bioprime Array CGH Genomic Labeling Module (Invitrogen, Carlsbad, CA). After labeling tumor DNA and reference DNA samples were pooled, cleaned and hybridization cocktails were prepared as instructed in the protocol. Hybridization and washes were performed according to the protocol using the human genome CGH 44B oligo microarrays (catalog number G4410-60520 by Agilent Technologies). A laser confocal scanner (Agilent Technologies) was used to obtain signal intensities from targets, and Feature Extraction software (version 8.1.1.1, Agilent Technologies) was applied in image analysis using manufacturer's recommended settings (44K_CGH_0605). To analyze the aCGH data we used CGH Analytics software (version 3.2.32, Agilent Technologies). Quality metrics provided by CGH Analytics were evaluated to ensure good data quality.

### Array CGH data visualisation

The visualisation tool called CGHviewer was written in Tcl/Tk. It was developed within the Gscope platform (Ripp et al., in preparation). The program CGHviewer is available as a Windows executable coupled with an installer/uninstaller (Contact: carles@igbmc.u-strasbg.fr). CGHviewer allows the import of text files. The human genome annotation that has been integrated into the current version of CGHviewer comes from the June 2005 assembly on the UCSC Genome Browser [23]. CGHviewer allows interactive graphical exploration of individual arrays or groups of arrays on genomic or chromosomic scales. It also allows the detection and visualisation of the BAC outliers. The visualisation of the aCGH data is provided before and after segmentation by CBS [15]. CGH log-ratio values are all displayed as log2. For the genomic displays, the plots consist of a x-axis divided into 24 chromosomes (22 chromosomes plus chromosomes X and Y). CGHviewer provides a zoom-in option and a view-finder. Pointing at a measurement in a plot shows the coordinates, and pointing at a box shows the "identity card" for BAC clones, oligonucleotide probes, genes and cytobands. The "identity card" includes information such as the accession number, the human genome position and for BACs and oligos, the log-ratio values.

### Statistical methods

The autocorrelation for BACs and oligos has been computed using the formula ∑i=14n−1(xi−μ)(xi+1−μ)σ2
 MathType@MTEF@5@5@+=feaafiart1ev1aaatCvAUfKttLearuWrP9MDH5MBPbIqV92AaeXatLxBI9gBaebbnrfifHhDYfgasaacH8akY=wiFfYdH8Gipec8Eeeu0xXdbba9frFj0=OqFfea0dXdd9vqai=hGuQ8kuc9pgc9s8qqaq=dirpe0xb9q8qiLsFr0=vr0=vr0dc8meaabaqaciaacaGaaeqabaqabeGadaaakeaadaaeWaqaamaalaaabaGaeiikaGIaemiEaG3aaSbaaSqaaiabdMgaPbqabaGccqGHsisliiGacqWF8oqBcqGGPaqkcqGGOaakcqWG4baEdaWgaaWcbaGaemyAaKMaey4kaSIaeGymaedabeaakiabgkHiTiab=X7aTjabcMcaPaqaaiab=n8aZnaaCaaaleqabaGaeGOmaidaaaaaaeaacqWGPbqAcqGH9aqpcqaIXaqmcqaI0aanaeaacqWGUbGBcqGHsislcqaIXaqma0GaeyyeIuoaaaa@49AB@. The autocorrelation measures the correlation in longitudinal data between a position *x*_*i *_and the next position *x*_*i*+1_. In this paper, *x*_*i *_and *x*_*i*+1 _correspond to consecutive BACs or oligos. *μ *and *σ *are respectively the mean and the standard deviation of the *n *log-ratios observed in a chromosome either for BAC or oligo aCGH. The lower the autocorrelation, the higher the noise.

Kendall's correlation is a ranks correlation measure defined by *τ *= 2*P*((*X*1 - *X*2)(*Y*1 - *Y*2) > 0) - 1. In case of independence between variables X and Y, *τ *follows the gaussian law *N*(0, 2(2n+5)9n(n+1)
 MathType@MTEF@5@5@+=feaafiart1ev1aaatCvAUfKttLearuWrP9MDH5MBPbIqV92AaeXatLxBI9gBaebbnrfifHhDYfgasaacH8akY=wiFfYdH8Gipec8Eeeu0xXdbba9frFj0=OqFfea0dXdd9vqai=hGuQ8kuc9pgc9s8qqaq=dirpe0xb9q8qiLsFr0=vr0=vr0dc8meaabaqaciaacaGaaeqabaqabeGadaaakeaadaGcaaqaamaalaaabaGaeGOmaiJaeiikaGIaeGOmaiJaemOBa4Maey4kaSIaeGynauJaeiykaKcabaGaeGyoaKJaemOBa4MaeiikaGIaemOBa4Maey4kaSIaeGymaeJaeiykaKcaaaWcbeaaaaa@3AFA@) *τ *varies between -1 and 1, and *τ *= 2*P*((*X*1 - *X*2)(*Y*1 - *Y*2) > 0) - 1 means that for two pairs of values (*X*1, *Y*1) and (*X*2, *Y*2), if *X*2 is greater (resp. smaller) than *X*1, then *Y*2 is greater (resp. smaller) than *Y*1 with a probability equal to *P*((*X*1 - *X*2)(*Y*1 - *Y*2) > 0).

The Circular Binary Segmentation method is a change point analysis accessible through the DNAcopy, v1.1.2 (R) library [15]. The parameters were chosen as follows: alpha = 0.01, number of permutations = 1000 and window size = 200 to increase the speed of the algorithm.

The R language was used for all statistical tests and plots except for the detection of BAC outliers.

## Abbreviations

BAC: Bacterial Artificial Chromosome; CGH: Chromosomal Comparative Genomic Hybridization; aCGH: array CGH; CBS: Circular Binary Segmentation

## Authors' contributions

The two first authors contributed equally to the work. IGM, MW, AV, JAS, DEN and OK collected patient samples and designed the experiments. NW, AC, HE, FB and BW analysed the data. NW and AC developed the statistical model and validated the BACs outliers. OP conceived the study and participated in the analysis. All authors read and approved the final manuscript.
